# Training, certification and accreditation for eye teams

**Published:** 2014

**Authors:** Karl Golnick, Lynn Anderson

**Affiliations:** President: Joint Commission on Allied Health Personnel in Ophthalmology; Director for Education: International Council of Ophthalmology.; CEO: Joint Commission on Allied Health Personnel in Ophthalmology. landerson@jcahpo.org

**Figure:**
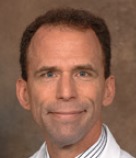
Karl Golnick

**Figure:**
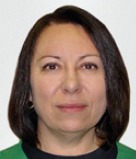
Lynn Anderson

Recent data suggests there may never be enough ophthalmologists to meet the needs of global eye care.^1^ One way to lessen the impact of the shortage is to increase the efficiency and effectiveness of each ophthalmologist by putting together a team of well trained personnel to support them. This increases both the availability and quality of eye care.

The ophthalmologist-led eye care team can include optometrists, nurses, mid-level ophthalmic personnel (ophthalmic assistants/technicians/medical technologists), refractionists, orthoptists, contact lens fitters, opticians, ophthalmic photographers, community health workers, low vision and rehabilitation specialists, and may include other community workers (e.g. teachers) who screen and refer potential eye patients.

Eye care teams may be large or small, depending on their goals and needs. In a teaching hospital, there will be teams of doctors, residents, nursing staff, allied health providers and the patient's primary care team, all of whom need to coordinate with each other. In an outpatient setting, the team may consist only of a doctor and mid-level ophthalmic personnel. Whatever the setting, team members should be performing their agreed-upon roles, allowing each team member to complete their assigned tasks, prevent duplication of effort, and maximise each team member's skills. This results in the ophthalmologist being able to care for the maximum number of patients by focusing her or his expertise on diagnosis and treatment.

**Strategies for team training**

**Cross training.** For example, residents can be asked to teach the nursing staff and mid-level ophthalmic personnel about diseases and new treatments. Nurses can be asked to show residents how to maximise the skills of a nurse oran ophthalmic surgical assistant in the operating theater. Ophthalmic assistants or technicians can capably and effectively train residents in the fundamentals of refraction and help them to refine their refraction skills.

**Grand rounds.** ‘Grand rounds’ sessions are case-based discussions and include all members of the team. They are designed to illustrate individual roles and responsibilities in providing efficient, effective patient care. A regular, internal training system using a ‘grand rounds’ method can effectively address the key knowledge and skills (or ‘core competencies’) needed by the eye care team. This can positively impact patient outcomes on an ongoing basis.

**Figure:**
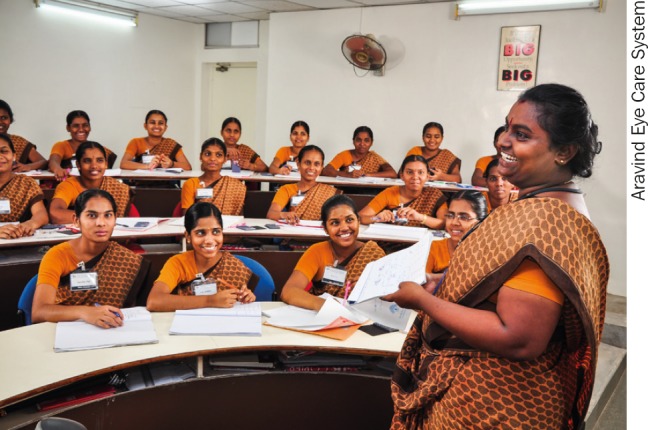
Eye care training programmes can be accredited to ensure they meet quality standards

Teamwork is a complex process but there are best practice principles for forming and using a team approach to improve access and quality of patient care. The team approach should be more than simply assembling the various team members required. It is crucial to define roles, responsibilities, job descriptions and sub-teams, and to promote team member cooperation.^2^ Good team communication is essential and should result in mutually agreed upon roles. Exact descriptions of these roles should be known and understood by all team members. The team should work together to make decisions and formulate goals. This approach will foster a sense of common purpose, create shared responsibility for team actions, and increase teamwork to achieve team goals. Cross-training (see panel) will improve the adaptability and flexibility of team members and ultimately improve team effectiveness. Finally, a well-functioning team will provide mechanisms for interactive conflict resolution.

The ability of each team member to perform her or his required function is essential to the team's success. Professional development, through individual certification and programme accreditation, aims to ensure that team members are competent in their roles.

**Certification** usually includes a standardised test of an individual's competence. Some countries already have certification processes for most eye care team members but many do not. The International Council of Ophthalmology (ICO) has international examinations to certify ophthalmology residents. The Joint Commission on Allied Health Care in Ophthalmology (JCAHPO) currently certifies several levels of allied health care in 29 countries. National and/or international certification should be developed for all eye care team members in so that their competence can be assured.

**Accreditation** is a process designed to ensure that training programmes meet quality standards. The accreditation process assesses the training programme based on standardised guidelines. Typically, the programme completes a self-assessment document that is then evaluated by the accrediting organisation. This is followed by a site visit in which an outside observer verifies compliance with accreditation guidelines. Some countries, but not all, have mechanisms in place to accredit eye care training programmes. Recently, international guidelines for ophthalmic allied health training programmes have been established by the International Joint Commission on Allied Health Care in Ophthalmology (IJCAHPO).^3^ India, Pakistan and Singapore have IJCAHPO-accredited programmes.

In summary, the high-performing eye care team is widely recognised as a fundamental tool for constructing a more patient-centred, coordinated, effective and high quality eye care delivery system. Having health care teams with well-defined roles and proof of competence is essential to meet global eye care needs. A well functioning ophthalmologist-led eye care team should increase efficiency and availability of care in a cost-effective manner and improve the health systems in a country.
